# Seeing the impossible: the impact of watching magic on positive emotions, optimism, and wellbeing

**DOI:** 10.7717/peerj.17308

**Published:** 2024-04-30

**Authors:** Richard Wiseman, Caroline Watt

**Affiliations:** 1 Department of Psychology, University of Hertfordshire, Hatfield, Hertfordshire, United Kingdom; 2 School of Philosophy, Psychology and Language Sciences, University of Edinburgh, Edinburgh, Midlothian, United Kingdom

**Keywords:** Psychology, Magic, Hope, Optimism, Impossible, Wellbeing, Illusion, Emotion

## Abstract

Watching a magic trick is a unique experience in which seemingly impossible events appear possible but without any suspension of disbelief. Unfortunately, relatively little work has examined the psychological impact of this fascinating experience. In the current study, participants first completed a measure of the degree to which they disliked magic (Loathing of Legerdemain Scale: LOLS) and then watched a video that either contained a series of magic tricks (magic video) or carefully matched non-magic tricks (control video). Participants then rated the degree to which they experienced positive epistemic emotions (Epistemically Related Emotion Scale: ERES), their belief about impossible events being possible in the future (Modal Judgment Task: MJT), general optimism (State Optimism Measure: SOM) and subjective wellbeing (Satisfaction With Life Scale: SWLS). Compared to participants who watched the control video, those who saw the magic video reported more positive epistemic emotions on the ERES. There were no significant differences on the MJT, SOM and SWLS. Participants’ LOLS scores were negatively correlated with the ERES, SOM and SWLS, suggesting that those who like magic are more likely to experience positive epistemic emotions, have higher levels of general optimism, and express greater satisfaction with their lives. These findings are discussed within the context of short-term and long-term exposure to magic, along with recommendations for future work.

## Introduction

A large body of research has explored the psychology of entertainment magic (for reviews see Kuhn, 2019; Lamont & Wiseman, 1999; Macknik, Martinez-Conde & Blakeslee, 2010). Most of this work has examined the principles underpinning conjuring tricks, including how magicians misdirect attention, encourage the erroneous perception of events, disrupt problem solving and manipulate memory. A smaller body of research has also explored the use of magic within applied settings (for reviews see Bagienski & Kuhn, 2019 and Wiseman, 2023), including how watching and learning tricks can help to overcome movement difficulties (*e.g.*, Green et al., 2013; Hines et al., 2018), reduce the perception of pain in dental and medical procedures (*e.g.*, Kothari et al., 2023; Labrocca & Piacentini, 2015; Thosar et al., 2022) and boost learning and knowledge retention (*e.g.*, Lustig, 1994; Moss, Irons & Boland, 2017).

Although some work has examined the neural activity associated with seeing magic (*e.g.*, Parris et al., 2009), little work has investigated the psychological impact of such experiences. This paucity of research is perhaps surprising given that a large body of work suggests that other seemingly impossible experiences can have a considerable and positive effect on individuals. For example, Wiseman & Watt (2022) reviewed work showing that a wide variety of seemingly impossible experiences (including engaging in fantasy play, reading science fiction, and having dreams that are comprised of impossible happenings) are associated with increased creativity. Similarly, other research has shown that some perceived paranormal experiences promote wellbeing and growth. For example, Palmer & Braud (2002) found that mystical experiences help to provide an enhanced sense of meaning and purpose, and Greyson (2013) reviewed studies showing that near-death experiences can decrease fear of death, increase self-esteem, and boost feelings of wonder. Finally, some work has explored how watching magic tricks presented in a variety of ways can change people’s attitudes to a range of seemingly impossible phenomena, including the existence of psychic ability, life after death and pseudo-psychological skills (for a review, see Lan et al., 2018).

Watching a magic trick is an especially interesting type of seemingly impossible experience because it is unique, easily produced, and frequently encountered in everyday life. On a theoretical level, several academics and performers have explored why watching a good magic trick is such an unusual, and potentially powerful, experience. Magic tricks involve two main components, the effect and the method. The effect is a seemingly impossible event (*e.g.*, the magician levitates above the stage) and the method is the secret part of the trick that makes the effect appear possible (*e.g.*, the concealed wires supporting the magician). Stromberg (2012) notes that Teller (half of the American duo Penn and Teller) describes magic as simultaneously real and unreal, and frames it as an odd, compelling and intellectual experience. Leddington (2016) argues that magic is a type of theatre that seems to present impossible events and at the same time to represent them as impossible. Finally, Lamont (2010) and Lamont (2017) notes that during a magic show audiences do not have to suspend their disbelief about the method. For example, in a Peter Pan play, the wires supporting the actors during the flying sequence might be obvious and the audience are required to implicitly ignore them, whereas in a magic show the wires supporting the magician need to be concealed. On a more pragmatic level, the experience is relatively common, with magicians performing in a wide variety of live settings (*e.g.*, theatres, cabarets, restaurants, bars, and parties), and producing recordings that frequently attract large audiences on both television and the Internet. The current study aimed to explore this topic, and to examine both the short-term and long-term impact of watching magic.

A small amount of work has assessed the short-term emotional impact of seeing videos of magic tricks. Magic tricks elicit a strong sense of cognitive dissonance (wherein a seemingly impossible event appears possible), and several studies have examined the degree to which they result in enhanced curiosity, surprise, and interest. For example, Ozono et al. (2021) had participants watch over a hundred short videos of magic tricks and rate whether they knew how each trick was achieved, along with their levels of three key epistemic emotions (surprise, interest, and curiosity). In general, being fooled by an illusion was positively related to each of these emotions. Subbotsky (2010) explored the impact of framing impossible events as either magic tricks or scientific phenomena and discovered that the former frame elicited a greater sense of exploratory behaviour and curiosity. Bagienski & Kuhn (2023) showed that the more impossible a magic trick appeared to be, the more people enjoyed watching it (although the same type of finding was not obtained by Kuhn et al. (2023). In similar work, Wiseman, Houstoun & Watt (2020) had participants rate how entertaining and interesting they found a video that used magic tricks to illustrate the Apollo Moon landings *versus* a near-identical control video not containing any magic tricks. Compared to the control video, participants rated the magic-based video as more entertaining and interesting. Because of this, it is expected that watching magic will make people more curious, surprised, and excited.

Other research has explored whether watching magic tricks provides a short-term boost to creativity and encourages a more expansive mindset. For example, Wiseman, Wiles & Watt (2021) had children complete a standard creativity measure (the Alternative Uses Test), either learn a magic trick or create an illusion-based drawing, and then complete the creativity test a second time. Compared to the children making the drawing, those watching the magic trick exhibited significantly higher scores on the second creativity test. Similarly, Haritaipan, Saijo & Mougenot (2018a) and Haritaipan, Saijo & Mougenot (2018b) had design students read about a variety of magical illusions and then create new designs for everyday objects. Knowing about the illusions resulted in more creative designs. Also, Li (2020) had design students complete the Alternative Uses Test both before and after learning about magic, and discovered that their creativity scores were significantly higher after the intervention. In other work, Subbotsky, Hysted & Jones (2010) had children undertake a standard creativity test, watch a film that either portrayed ‘magical’ events or non-magical actions, and then carry out the test again. Participants viewing the ‘magical’ film clip obtained higher scores on the second creativity test. Finally, Wiseman, Houstoun & Watt (2020) noted that participants were more likely to report believing that anything was possible after watching a video containing magic tricks compared to a control video.

In related work, some magicians have argued that watching magic creates a more expansive mindset. For example, illusionist David Copperfield has noted how his shows may encourage audiences to believe that the seemingly impossible might be possible in the future (Morehart, 2021) and Marco Tempest has described magic as a way of ‘prototyping the future’ (Royal Society of the Arts, 2013). In line with this notion, some researchers have explored how experiencing the seemingly impossible might make people more optimistic about other apparently impossible events becoming a reality in the future. For example, Black, Capps & Barnes (2018) explored people’s familiarity with science fiction and their beliefs about the impossible. Participants indicated whether they recognised authors from various literary genres and then completed the Modal Judgment Task (MJT) in which they rated whether they believed that a series of seemingly impossible events (*e.g.*, telepathy and time travel) might be possible in the future. Greater knowledge of (and therefore presumably exposure to) science fiction authors was positively related to higher scoring on the MJT. Based on this work, it was expected that watching magic may make people more open to the possibility of seemingly impossible events being possible in the future, and boost their general levels of optimism (defined as a general expectation that future events will work out well).

The results from a relatively large body of research suggests that watching more traditional performing arts (such as drama, dance, and music) boosts happiness and life satisfaction. For example, several surveys have discovered a positive correlation between theatre attendance, happiness, and life satisfaction (*e.g.*, Michalos & Kahlke, 2010; Wheatley & Bickerton, 2017; Hand, 2018), and others have used experience sampling techniques and suggest that watching a play, dance, or concert considerably enhances short-term happiness (*e.g.*, Bryson & MacKerron, 2017). Based on this work, it was expected that watching magic would be associated with greater life satisfaction.

Other work has studied the longer-term impact of seemingly impossible experiences by examining the relationship between a lifetime exposure to such phenomena and various measures (Black, Capps & Barnes, 2018; Wu, Zhou & Zhang, 2022). Although no previous work has explicitly examined the correlates of long-term exposure to magic, work by Silvia et al. (2023) can be seen as informing this issue. During this research, four groups of adult participants completed a short scale designed to measure a general dislike for watching magic (the Loathing of Legerdemain Scale: LOLS) along with a bank of other questionnaires. The work yielded several sets of findings. First, on an emotional level, participants who disliked magic had a lower propensity for curiosity, awe, wonder and absorption (as measured by Openness to Experience and the Unusual Aesthetic Experiences scales). Second, it was predicted that those who disliked magic would be uncomfortable not knowing the secrets to illusions and so have a higher need for certainty and cognitive structure. This was confirmed by significant correlations between the LOLS and the Brief Dogmatism Scale and Intolerance of Uncertainty Scale. Third, as magic is often seen as a form of manipulation, it was predicted that people who disliked watching tricks would have a stronger need to control others. This was confirmed by strong correlations between the LOLS and the Dominance-Prestige Scale. There was also evidence that those who disliked magic exhibited higher levels of psychopathy (the 12-item Dirty Dozen Scale), and that participants with less faith in humanity tended to dislike magic. The correlational nature of this work means that it is not possible to know if disliking magic results in these attributes, if individuals with such attributes are more likely to dislike magic, or a combination of the two mechanisms.

The current study is an initial attempt to examine both the short-term and long-term impact of observing magic. The former involves participants completing questionnaires after either watching a video containing magic tricks or a control video. The latter assumes that the more a person reports liking magic, the more likely they are to seek out opportunities to watch magic, and involves examining the relationship between scores on the LOLS and the questionnaires. Participants first completed the LOLS, and then saw a video of six short magic tricks (magic video) or six non-magic tricks (control video). The non-magic tricks were carefully matched to the tricks to ensure that they contained the same apparatus and actions, but did not show any seemingly magical events. Participants then completed a shortened version of the Epistemically Related Emotion Scale (ERES) measuring three positive epistemic emotions: surprise, excitement, and curiosity. They were then presented with a version of the Modal Judgment Task (MJT) and asked to rate whether they believed that a series of seemingly impossible events might be possible in the future. Finally, participants completed questionnaires evaluating their general optimism (State Optimism Measure: SOM) and subjective wellbeing (Satisfaction With Life Scale: SWLS).

It was predicted that participants’ scores on the ERES, MJT, SOM and SWLS would be significantly higher after watching the magic video compared to the control video, and that all these measures would be significantly and negatively correlated with the LOLS.

## Method

Participants (*n* = 200, mean age = 39.20 years, *SD* = 13.90; range 19–72 years) were recruited from the crowdsourcing platform, Prolific Academic (https://www.prolific.com/). Several studies have validated the use of these types of platforms in psychological research (*e.g.*, Crump, McDonnell & Gureckis, 2013; Enochson & Culbertson, 2015). Estimating a prior expected effect size was not possible to due to the paucity of past research, however, the predetermined sample size had a good chance of detecting a medium sized effect (*d* = 0.5, *p* < 0.05, 2-tailed, power = 0.9).

## Materials

### Stimulus videos

Both the magic video and the control video featured the first author, who has been performing magic for over 40 years and has worked as a professional magician. The magic video lasted 2 min 15 s, contained six short tricks, and showed the upper body and hands of the performer. The six tricks were chosen to be easy to understand, highly visual and frequently performed by magicians. The video contained the following six tricks:

(1) The magician shows two sponge balls and places one in each of their fists. When the hands are opened, a ball has vanished from one hand and appeared in the other;
(2) The magician draws an X on a playing card and puts the card into the centre of the pack. It then reappears on the top of the deck;
(3) A red handkerchief is placed into the fist and vanishes;
(4) A playing card is folded in half lengthways so that viewers can only see the back of the card. The card then appears to magically reverse itself, such that it is still folded in half but the face is showing;
(5) Several coins are placed into a small metal box and the box is put on the back of the hand. The coins appear to penetrate through the box and the hand;
(6) A rope is cut in half and the two ends tied together. The rope appears to become restored into a single rope.

The control video lasted the same amount of time as the magic video, contained six events, and again showed the upper body and hands of the performer. The six events involved the same apparatus and actions as the magic video, but did not involve any surprising events. The video contained the following six events:

(1) The magician places a sponge ball in each hand and the balls remain in each hand;
(2) The magician draws an X on a playing card and puts the card into the centre of the pack. The card remains in the centre of the deck;
(3) A red handkerchief is placed into the hand and remains there;
(4) A playing card is folded in half lengthways so that only the back of the card is visible, and does not magically reverse itself;
(5) Several coins are placed into a small metal box and the box is put on the back of the hand. The coins remain in the box;
(6) A rope is cut in half and the two ends tied together. The two pieces of rope are not restored.

#### Loathing of Legerdemain scale (Silvia et al., 2023)

Participants rate four statements about their attitude towards magic (*e.g.*, ‘I find magic tricks annoying’) on a 5-point scale between 1 (Strongly disagree) and 5 (Strongly agree). Higher scores indicated a stronger dislike of magic.

#### The epistemically related emotion scale (Pekrun et al., 2017)

The study utilised the three sub-scales that relate to positive epistemic emotions (surprise, curiosity, excitement), with each subscale involving participants rating the degree to which they have just experienced three named emotions (*e.g.*, ‘Joyful’ or ‘Amazed’) on a 5-point scale between 1 (Not at all) and 5 (Very strong). Higher scores indicated stronger emotional responses.

#### Modal judgment task (Black, Capps & Barnes, 2018)

Participants are asked about the likelihood of nine seemingly impossible events happening in the future (*e.g.*, ‘Will it ever be physically possible for humans to transform into some type of animal?’) and respond to each item by selecting either ‘Yes’ or ‘No’. This measure was devised for a study that examined the effects of being exposed to science fiction, and included items about a person transforming into an animal, communicating using telepathy, moving through rock by changing their atomic structure, travelling through time, becoming an Android, and shielding the Earth from alien attack. Responses were summed (Yes =1, No =0) so higher scores indicated greater belief in seemingly impossible events being possible in the future.

#### State optimism measure (Millstein et al., 2019)

Participants are presented with seven statements about how optimistic they feel at that moment (*e.g.*, ‘I am feeling optimistic about life’s challenges’), and rate each statement on a 5-point scale between 1 (Strongly disagree) and 5 (Strongly Agree). Higher scores indicated greater levels of perceived optimism.

#### The satisfaction with life scale (Diener et al., 1985)

Participants see five items about their general life satisfaction (*e.g.*, ‘In most ways my life is close to my ideal’) and rate each statement on a 7-point scale from 1 (Strongly disagree) to 7 (Strongly agree). Higher scores indicated greater levels of perceived life satisfaction.

The authors have permission to use all the above instruments from the copyright holders.

## Procedure

The study was approved by the University of Hertfordshire Health, Science, Engineering & Technology Ethics Committee (number aLMS/SF/UH/05334(1)). Participants were recruited on the Prolific Academic (https://www.prolific.com/) crowdsourcing platform, and the study was presented *via* the Qualtrics platform (https://www.qualtrics.com/). After providing written consent, participants were asked to enter their age and to complete the Loathing of Legerdemain Scale (LOLS). They were then randomly assigned to watch either the magic video or the control video. Those watching the magic video were told that they would see some magic tricks and those watching the control video were told that they would see someone playing with some objects. After seeing one of these videos, participants completed the Epistemically Related Emotion Scale (ERES), the MJT, the SOM and the SWLS. The time taken for each participant to complete the survey was recorded and they received a small monetary reward for taking part.

## Results

There were 101 participants in the magic video condition, and 99 in the control video condition. All participants, conditions, measures and data were reported and included in the analyses. The groups did not differ in age (magic video = 39.10 years, *SD* = 14.09; control video = 39.32, *SD* = 13.69; *t*_198_  = −0.12, *p*  = 0.91), LOLS score (magic video = 8.06, *SD* = 3.27; control video = 7.72, *SD* = 3.00; *t*_198_  = .77, *p*  = 0.4), or time taken to complete the study (magic video = 504.71 s, *SD* = 245.06; control video = 491.69 s, *SD* = 318.22; *t*_198_  = 0.32, *p*  = 0.75).

Unpaired *t*-tests compared the scores obtained from the two groups on the ERES, MJT, SOM and SWLS. Compared to the control video, the magic video was rated as significantly more surprising, curious, and exciting. There was no difference between the two groups on the MJT, SOM and the SWLS (see Table 1).

**Table 1:** Summary data for the magic video compared to the control video.

Scale	Magic Video	Control Video			
	*M* (*SD*)	*M* (*SD*)	*t*	*p*	*d* [95% CI]
ERES - Surprise	2.85 (1.02)	1.73 (.68)	9.15	**.00001**	1.30 [.98, 1.59]
ERES - Curiosity	3.82 (.94)	2.71 (.96)	8.25	**.0001**	1.17 [.87, 1.47]
ERES - Excitement	3.00 (1.06)	2.35 (.96)	3.82	**.0002**	.54 [.36, .93]
MJT	14.58 (1.89)	14.53 (2.13)	.17	.86	.02 [−.25, .30]
SOM	23.99 (6.64)	24.01 (5.90)	−.02	.98	−.003 [−.28, .27]
SWLS	20.01 (7.59)	20.12 (7.14)	−.11	.91	−.01 [−.29, .26]

**Notes.**

The items on all the scales were reverse scored where necessary and summed. Means, *SD* s (in parentheses), unpaired *t*-values (*df* = 198), *p*-values (2-t; significant *p*-values in bold), and effect sizes (Cohen’s *d*; 95% Confidence Intervals in brackets) for participants watching the magic Video (*n* = 101) compared to the control Video (*n* = 99).

Pearson correlations were calculated between participants’ LOLS scores and each of the variables (see Table 2). There were negative correlations between the LOLS and the ERES, SOM and SWLS. For completeness, Table 2 also contains additional analyses showing the correlations in each of the two conditions.

**Table 2:** Correlations between participants’ LOLS scores and each of the variables for the magic video, the control video, and overall.

Scale	Overall (*n* = 200)	Magic video (*n* = 101)	Control video (*n* = 99)	Magic *vs* control correlations
	*r* [95% CI] (*p*)	*r* [95% CI] (*p*)	*r* [95% CI] (*p*)	*z* (*p*)
ERES—Surprise	−.26 [−0.38, −0.13] **(.0002)**	−.50 [−0.63, −0.34] **(.0001)**	−.12 [−0.31, 0.079] (.28)	2.98 **(.003)**
ERES—Curiosity	−.28 [−0.41, −0.15] **(.0001)**	−.58 [−0.7, −0.43] **(.0001)**	−.12 [−0.31, 0.079] (.25)	−3.77 **(.0002)**
ERES—Excitement	−.39 [−0.5, −0.26] **(.0001)**	−.61 [−0.72, −0.47] **(.0001)**	−.18 [−0.36, 0.018] (.07)	−3.67 **(.0002)**
MJT	−.017 [−0.16, 0.12] (.82)	−.09 [−0.28, 0.11] (.36)	.06 [−0.14, 0.25] (.58)	−1.05 (.29)
SOM	−.17 [−0.3, −0.032] **(.02)**	−.18 [−0.36, 0.016] (.08)	−.16 [−0.35, 0.039] (.11)	−0.14 (.89)
SWLS	−.16 [−0.29, −0.022] **(.03)**	−.17 [−0.35, 0.026] (.09)	−.14 [−0.33, 0.059] (.16)	−0.21 (.83)

**Notes.**

Pearson correlations between participants’ LOLS scores and other variables (95% Confidence Intervals in brackets; 2-t *p*-values in parentheses; significant *p*-values in bold). For completeness, the correlations are also shown for each of the two conditions, along with the *z*-score and associated 2-t *p*-values comparing the correlations in each condition.

## Discussion

To investigate the psychological impact of watching a magic trick, participants first completed a measure of their liking of magic (LOLS) and then watched a video that either contained a series of magic tricks (magic video) or a carefully matched series of non-magic tricks (control video). They then completed measures relating to positive epistemic emotions (ERES: surprise, curiosity, and excitement), attitudes towards seemingly impossible events (MJT), general optimism (SOM) and life satisfaction (SWLS). Each of the findings will be discussed in turn.

One key part of the study examined the short-term impact of watching magic by comparing the scores of participants who had watched the magic video with those who had seen the control video.

First, compared to the control video, watching the magic video yielded higher scores on the ERES measures of surprise, curiosity, and excitement. This is consistent with previous work on the observation of magic (Ozono et al., 2021; Wiseman, Houstoun & Watt, 2020). Future research could build on these findings by developing a more fine-grained understanding of the emotional response to magic, perhaps focusing on identifying what (if anything) is unique about the emotions associated with watching the seemingly impossible made possible. Experiencet seems likely that this work may benefit from, and contribute to, recent advances in the psychology of awe, wonder and astonishment (*e.g.*, Keltner, 2023; Shiota, 2021).

Second, compared to the control video, the magic video did not result in increased scoring on the MJT, SOM and SWLS. This could be because watching magic does not have a significant impact on people’s attitudes towards the seemingly impossible, general optimism, or satisfaction with life. Alternatively, the lack of impact may be due to the stimuli used in the study. Compared to the type of magic performances that people typically encounter in everyday life, the magic video was short and contained relatively basic tricks. Future work could overcome these issues by, for example, employing longer videos and including tricks that are more impactful. It has been widely argued that videos of magic tricks do not have the impact of a live performance, perhaps because the effects seen in videos could be the result of camera trickery (*e.g.*, Olson, Demacheva & Raz, 2015). As a result, future research could also utilise live performances as stimuli (*e.g.*, Bagienski & Kuhn, 2023) or film the tricks in front of an audience to help assure viewers that the illusions were genuine. In addition, such studies could involve participants rating the perceived impossibility of the trick, along with whether they have seen it before and believe that they know how it was achieved.

Third, when considering the impact of watching magic on beliefs about seemingly impossible events, future research might want to revisit the items in the MJT. This measure was devised to reflect the type of impossible events that occur in science fiction books, television shows and films (*e.g.*, building a computer more powerful than the mind and shielding the Earth from alien attack). As a result, the MJT contained several items that were not directly related to the type of impossible events shown in the magic video. Future work may benefit from developing items that are associated with the type of effects routinely produced by magicians, such as the sudden disappearance of an object, or the transformation of one object into another.

Fourth, additional work could explore the types of individual differences that may come into play when people watch magic. For example, when observing a trick some people focus on trying to figure out the secret to the illusion whereas others enjoy experiencing the impossible events that appear to be happening (*e.g.*, Gronchi & Zemla, 2021; Silvia et al., 2023). In addition, Black & Barnes (2021) discovered that the degree to which participants felt transported or absorbed in an experience determined the degree to which watching a science fiction television programme boosted their scores on a creativity task. It seems likely that both these factors will significantly interact with the way in which people perceive, and are impacted by, magic. Future studies may benefit from exploring these, and other, individual differences.

A second major part of the present study examined the long-term impact of watching magic by examining the correlations between participants’ dislike of magic (LOLS scores) and the other measures. Previous work showed a significant negative relationship between the LOLS and a general propensity to experience curiosity, awe, wonder and absorption (Silvia et al., 2023). Consistent with this, in the present study participants’ LOLS scores were significantly and negatively correlated with ERES overall measurements of excitement, curiosity, and surprise. Further analyses showed that these correlations were much higher among participants who had watched the magic video rather than the control video. Together, this work suggests that liking magic is related to a propensity to experience these types of positive emotions, and that watching magic can be a quick and effective catalyst for the emergence of such feelings. Future work could attempt to tease apart correlation from causation by examining, for example, whether liking magic (perhaps along with other stimuli designed to elicit a sense of wonder and awe) enhances the likelihood of experiencing such emotions, or whether those who naturally feel these emotions end up being attracted to magic, or a combination of the two mechanisms. This work could also explore the role that watching magic plays in this process.

LOLS scores were also significantly and negatively correlated with higher scores on the SOM and SWLS. This former finding is in line with the suggestion that exposure to magic results in a more expansive mindset and may make people more optimistic about the future (*e.g.*, Morehart, 2021; Royal Society of the Arts, 2013). The latter finding supports other survey-based work showing that watching many forms of performing arts boosts life satisfaction and happiness (*e.g.*, Michalos & Kahlke, 2010; Wheatley & Bickerton, 2017; Hand, 2018). Again, future work could examine the causal nature of these relationships. Findings suggesting that watching magic has a long-term and positive effect on optimism and life satisfaction, could pave the way for innovative magic-based interventions for happiness and wellbeing.

The present study has also helped to highlight some general issues related to future work in this area. First, such research may benefit from creating a more focused measure of magic exposure and appreciation. The LOLS was designed to reflect the degree to which participants dislike magic in general. In contrast, a more nuanced and fine-grained instrument could examine, for example, how often people see magic, the type of magic they especially enjoy watching, and whether they prefer to watch magic live or on video. Second, future work could expand the focus of the psychological impact of watching magic to include important social factors, such as how the experience provides people with a shared experience and helps them to bond with others.

## Conclusions

In summary, this study was designed to add to the small amount of work that has studied the short-term and long-term psychological impact of watching magic in everyday life. It has yielded several promising findings. Watching the magic video proved to be a quick and effective way of promoting three important and positive emotions: curiosity, surprise, and excitement. From a longer-term perspective, a general liking for magic was positively associated with both greater levels of general optimism and higher life satisfaction. It is hoped that this study will encourage others to carry out work into the topic, including developing a more detailed understanding of people’s emotional reaction to magic tricks, exploring the role that individual differences play in this process, building a more fine-grained measure of magic exposure and appreciation, identifying the social impact of observing magic shows, and creating effective magic-based interventions.

## Supplemental Information

10.7717/peerj.17308/supp-1 Supplemental Information 1 Dataset of condition, participant’s time, age, LOLS, MJT, SOM, ERES and SWLS Each row contains the score on each of the scales and measurements for each participant.

## Additional Information and Declarations

### Data Availability

The following information was supplied regarding data availability: The raw data for all of the scales is available in the Supplemental File.
